# Branch retinal artery occlusion with non-obstructive general angioscopy confirmed puff-chandelier rupture releasing cholesterol crystals in the aortic arch: a case report

**DOI:** 10.1093/ehjcr/ytae238

**Published:** 2024-05-10

**Authors:** Shohei Migita, Keisuke Kojima, Yasuo Okumura

**Affiliations:** Division of Cardiology, Department of Medicine, Nihon University School of Medicine, Ohyaguchi-kamicho, Itabashi-ku, Tokyo 173-8610, Japan; Division of Cardiology, Department of Medicine, Nihon University School of Medicine, Ohyaguchi-kamicho, Itabashi-ku, Tokyo 173-8610, Japan; Division of Cardiology, Department of Medicine, Nihon University School of Medicine, Ohyaguchi-kamicho, Itabashi-ku, Tokyo 173-8610, Japan

Retinal artery occlusion (RAO) typically occurs as a result of embolization from various potential sources including the ipsilateral internal carotid artery, aortic arch, and the heart. However, direct visualization of the embolic source is uncommon.

A 64-year-old man visited our hospital as a result of vision loss and was diagnosed with branch RAO following an ophthalmologic examination (*Panels A* and *B*). He had untreated hypertension and dyslipidaemia, silent sporadic bilateral cerebral infarctions on brain magnetic resonance imaging, and severe stenosis of coronary arteries on coronary computed tomography, without intracardiac thrombus or internal carotid artery stenosis. Coronary angiography showed a multivessel lesion, and non-obstructive general angioscopy (NOGA) showed puff-chandelier rupture (PCR) in the aortic arch (*Panels C* and *D*). Blood was sampled near the PCR, and its analysis via polarized light microscopy using the filter paper rinse method revealed cholesterol crystals (CCs; *Panels E* and *F*).

Non-obstructive general angioscopy allows for the direct visualization of vulnerable plaques in the aortic intima. Cholesterol crystals are found in PCR, a representative type of vulnerable plaque, and have been shown to cause embolism like stroke and induce inflammatory cytokines. In this case, CCs were detected from the PCR of the aortic arch, suggesting the possibility of atherothrombotic occlusion in the smaller arteries that control the ocular circulation through the internal carotid and ocular arteries. Cholesterol embolization syndrome is a common manifestation of advanced atherosclerosis. In our case, the patient was started on strict lipid control with a strong statin, to stabilize the vulnerable plaque in the aorta.

Fundus photography images of the retina of the right eye (*Panel A*) and the left eye (*Panel B*). In the left eye, occlusion of the branch retinal artery was observed. Observations of the aortic arch wall via non-obstructive general angioscopy and the white dotted line indicate the aortic arch wall (*Panel C*). The puff-chandelier rupture was observed by non-obstructive general angioscopy in the aortic arch (*Panel D*). Blood was sampled from around the puff-chandelier rupture observed in the aortic arch, and cholesterol crystals were observed under polarized light microscopy (*Panels E* and *F*).

**Figure:**
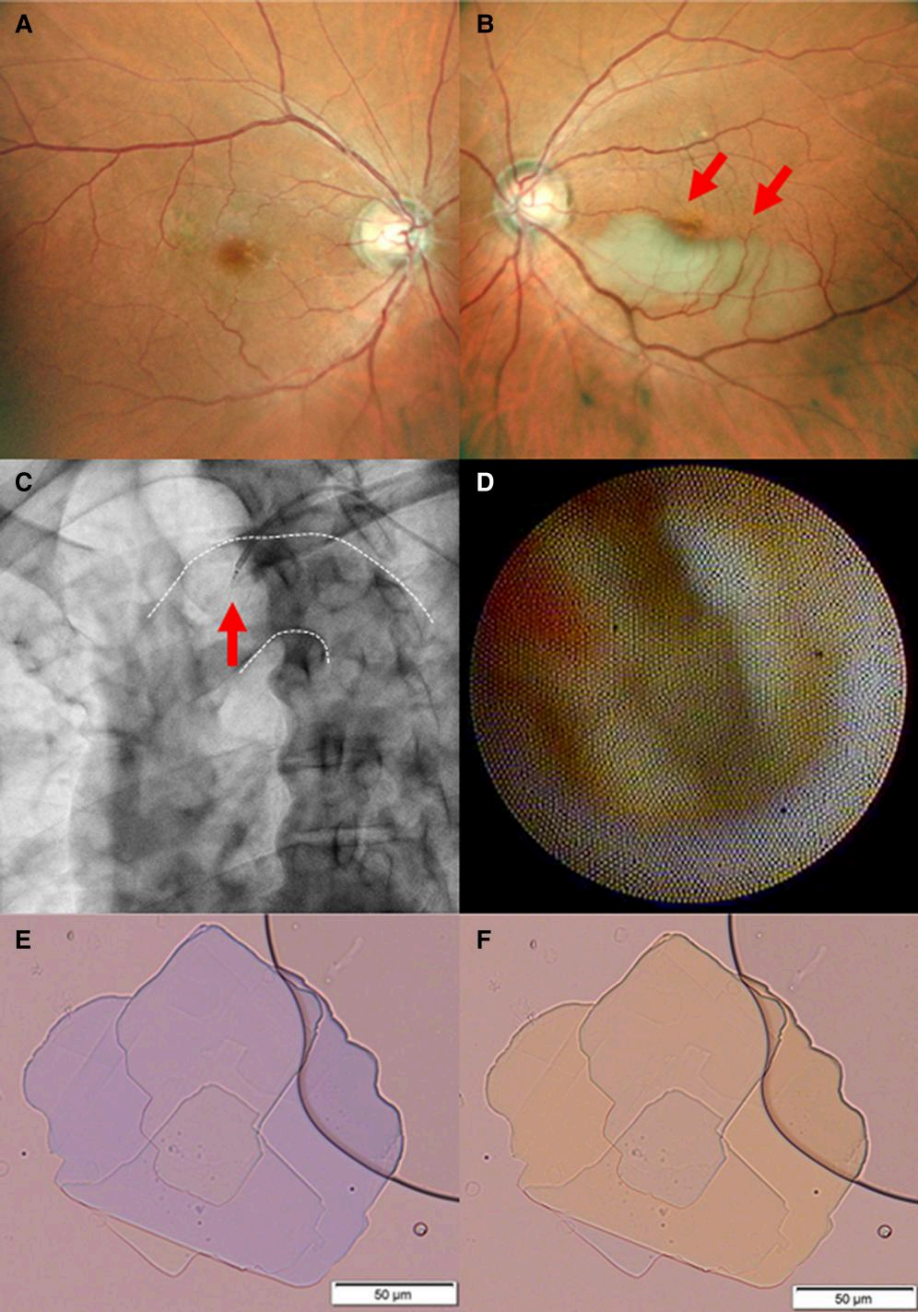


## Data Availability

The data underlying this article will be shared upon reasonable request to the corresponding author.

